# BiPAP for treating moderate and severe asthma exacerbations in a PED

**DOI:** 10.1186/cc12204

**Published:** 2013-03-19

**Authors:** A Williams, T Abramo

**Affiliations:** 1 Vanderbilt University Medical Center, Nashville, TN, USA

## Introduction

BiPAP utilization for the treatment of severe refractory status asthmaticus patients has become an accepted therapy but is not well described for moderate exacerbations. We sought to analyze outcomes from our BiPAP quality database for children presenting in status asthmaticus at varying levels of severity.

## Methods

PED status asthmaticus patients requiring BiPAP from 1 January 2010 to 31 August 2012 had a bedside interview and documentation of information at the time therapies were given. Incomplete data were collected retrospectively. All data were stored and analyzed using a RedCap database. Subjects were stratified into severity groups based on asthma score at the time of BiPAP placement.

## Results

**Figure 1:**
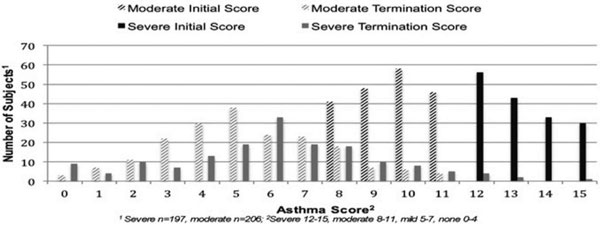
**Initiation and termination asthma scores grouped by severity**.

There were 206 subjects in the moderate severity group and 197 in the severe group. Table 1 shows the groups were well matched and compares other pertinent data. Children with severe presentations were placed on BiPAP sooner (*P <*0.001) and remained on BiPAP longer (*P <*0.001). The moderate group had a longer wait until BiPAP placement. Tables 2 and 3 demonstrate higher initial BiPAP (IPAP/EPAP) settings with increasing age and severity. Figure 1 trends initiation and termination asthma scores stratified by severity at BiPAP placement. More of the severe group was admitted to the PICU andhad overall longer hospitalizations (*P <*0.06). None experienced severe complications.

**Table 1:** Comparison of children receiving BiPAP for status asthmaticus by severity of illness

	Moderate (n = 206)	Severe (n = 197)	*P *value
Age (years)	6.8 ± SD 2.8	7.1 ± SD 4.2	X
Gender	62% male	61% male	X
History of asthma	92%	87%	X
Prior PICU admission	21%	32%	X
Prior history of BiPAP use	19%	24%	X
Prior history of intubation	6%	7%	X
Mean triage for BiPAP start (hours)	2.8 ± SD 2.1	1.2 ± SD 1.25	<0.0001
Mean time on BiPAP (hours)	3.9 ± SD 3.6	5.6 ± SD 5.2	<0.0001
Median time on BiPAP (hours)	2.7	3.8	X
Admission to PICU	58%	75%	X
Total length of hospital stay	42 ± SD 26	50 ± SD 33	<0.06
Serious complications	None	None	X
72-hour returns	n = 1 (0.5%) - no admits	n = 4 (2%) - no admits	X

**Table 2:** Mean settings by age: moderate group

Age (years)	IPAP	EPAP
<3 (*n *= 22)	12.1 ± SD 2.1	6.4 ± SD 1.3
3 to 4 (*n *= 48)	12.1 ± SD 1.6	6.4 ± SD 1.2
5 to 6 (*n *= 51)	13.0 ± SD 2.3	6.6 ± SD 1.3
7 to 8 (*n *= 24)	13.9 ± SD 2.1	7.2 ± SD 1.2
9 to 10 (*n *= 28)	13.9 ± SD3.2	7.4 ± SD 2.0
11 to 12 (*n *= 25)	13.8 ± SD 1.8	7.0 ± SD 1.3
13 to 14 (*n *= 10)	13.8 ± SD 3.2	7.4 ± SD 1.4
15 to 17 (*n *= 8)	17.0 ± SD 2.4	9.5 ± SD 0.9

**Table 3:** Mean settings by age: severe group

Age (years)	IPAP	EPAP
<3 (*n *= 21)	12.7 ± SD 2.0	6.8 ± SD 1.3
3 to 4 (*n *= 50)	13.3 ± SD 2.8	6.8 ± SD 1.4
5 to 6 (*n *= 26)	13.9 ± SD 2.2	7.3 ± SD 1.3
7 to 8 (*n *= 29)	15.0 ± SD 2.4	7.7 ± SD 1.5
9 to 10 (*n *= 26)	15.7 ± SD 3.5	8.0 ± SD 1.8
11 to 12 (*n *= 22)	14.5 ± SD 3.2	7.6 ± SD 1.6
13 to 14 (*n *= 9)	15.3 ± SD 3.2	8.0 ± SD 1.7
15 to 17 (*n *= 14)	17.5 ± SD 3.0	9.5 ± SD 1.8

## Conclusion

BiPAP is a benefi cial therapy for children presenting to the PED with severe asthma exacerbations. It may have utility for less severe asthma exacerbations.

